# Intermittent High Glucose Exacerbates A-FABP Activation and Inflammatory Response through TLR4-JNK Signaling in THP-1 Cells

**DOI:** 10.1155/2018/1319272

**Published:** 2018-04-11

**Authors:** Hui Li, Han-Ying Luo, Qing Liu, Yang Xiao, Lin Tang, Feng Zhong, Gan Huang, Jun-Mei Xu, Ai-Min Xu, Zhi-Guang Zhou, Ru-Ping Dai

**Affiliations:** ^1^Department of Anesthesiology, The Second Xiangya Hospital, Central South University, Changsha, China; ^2^Department of Metabolism and Endocrinology, The Second Xiangya Hospital, Central South University, Changsha, China; ^3^Department of Pharmacology & Pharmacy, Li Ka Shing Faculty of Medicine, The University of Hong Kong, Pokfulam, Hong Kong

## Abstract

**Background:**

Glucose fluctuation confers additional risks on diabetes-related vascular diseases, but the underlying mechanisms are unknown. Macrophage activation mediated by TLR4-JNK signaling plays an important role during the progress of diabetes. In the present study, we hypothesize that glucose fluctuation results in macrophage inflammation through TLR4-JNK signaling pathways.

**Methods:**

THP-1 cells were treated with normal glucose (5 mM), constant high glucose (25 mM), and intermittent high glucose (rotation per 6 h in 5 mM or 25 mM) for 24 h. The mRNA and protein expression levels of TLR4, p-JNK, and adipocyte fatty acid-binding protein (A-FABP) were determined, and the proinflammatory cytokines TNF-*α* and IL-1*β* were quantified.

**Results:**

In constant high glucose, TLR4 expression and JNK phosphorylation levels increased, and this effect was more pronounced in intermittent high glucose. Accordingly, the expression of A-FABP and the release of the proinflammatory cytokines TNF-*α* and IL-1*β* also increased in response to constant high glucose, an effect that also was more evident in intermittent high glucose. The inhibition of p-JNK by SP600125 did not attenuate TLR4 expression, but totally inhibited both A-FABP expression and the production of the proinflammatory cytokines TNF-*α* and IL-1*β* in both constant and intermittent high glucose.

**Conclusions:**

Intermittent high glucose potentiates A-FABP activation and inflammatory responses via TLR4/p-JNK signaling in THP-1 cells. These findings suggest a more detrimental impact of glucose fluctuation on macrophage inflammation in diabetes-related vascular diseases than thus far generally assumed.

## 1. Introduction

Accumulating evidence from epidemiological and interventional research has pointed toward the deleterious impact of hyperglycemia on the progress of diabetic complications [1–3]. However, more recent clinical studies have shown that glucose fluctuation plays a more important role in conferring additional risks on micro- and macrovascular diabetic complications [4–6] than merely high glucose levels do. In a study following 566 elderly patients with type 2 diabetes mellitus, glucose instability measured as the coefficient of variation of fasting plasma glucose concentrations for 3 years was found to independently correlate with cardiovascular-related mortality [7]. Glucose fluctuation not only exists in and accelerates the disease progress of diabetes, but also impairs the recovery of coexistent diseases [8, 9]. In a study of sepsis, the glycemic lability index was reported to be the best indicator to predict mortality, even after adjustment for confounders including the number of organ failures and hypoglycemia [8]. In addition, daily glucose fluctuation was also shown to affect vessel healing in coronary artery disease patients who had received everolimus-eluting stent implantation [10]. In spite of the fact that abundant clinical literature has depicted a critical role of glucose fluctuation in disease progress, irrespective of diabetes, the underlying mechanism by which glucose fluctuation exacerbates the disease progress remains unclear so far.

The macrophage, an inflammatory cell essential for the initiation and development of inflammation, is a key element in hyperglycemia-induced damage [11]. Current studies on macrophages focus predominantly on the impairment by consistent hyperglycemia. In primary human monocyte-derived macrophages, hyperglycemia induces the production of the prototype M_1_ cytokines tumor necrosis factor-*α* (TNF-*α*), interleukin-1*β* (IL-1*β*), and IL-6, but inhibits the M_2_ cytokines IL-1Ra and C-C motif chemokine ligand 18 (CCL18) during macrophage differentiation [12]. High glucose levels have been shown to markedly repress the expression of silent mating type information regulation 2 homolog 1 (SIRT1) and promote the release of proinflammatory cytokines in RAW264.7 macrophages [13]. While extensive research has been carried out on the effects of fluctuating hyperglycemia in patients with diabetes, coronary artery disease, and sepsis, few studies have examined the effect of fluctuating hyperglycemia on macrophage functioning. In this regard, previous studies in rodents reported that repetitive glucose fluctuation evoked more monocyte adhesion to the endothelium than stable glucose [14, 15].

Adipocyte fatty acid-binding protein (A-FABP, also referred to as FABP4, aP2) is highly expressed in adipocytes and macrophages and is extensively involved in inflammation and glucose and lipid metabolism [16]. A-FABP mainly functions as an intracellular transport protein for fatty acids, thereby regulating cellular lipid metabolism and lipid signals [17]. In macrophages, several proinflammatory stimuli have been reported to upregulate A-FABP expression, including oxidized low-density lipoproteins, toll-like receptor (TLR) agonists, and PPAR*γ* agonists [18–20]. In this regard, lipopolysaccharide (LPS), a TLR4 ligand, stimulates A-FABP expression and the activated A-FABP, reciprocally, enhances the LPS-TLR4 signaling-evoked c-Jun N-terminal kinase (JNK) inflammatory pathways [21]. In addition, prolonged hyperglycemia has been shown to induce A-FABP expression in mesangial cells and trigger the release of proinflammatory cytokines [22]. The concentration of A-FABP in circulation was found to strongly associate with diabetes, atherosclerosis, and coronary heart disease; it has been identified as a good biomarker in the prediction of the development of both diabetes and metabolic syndrome [23]. However, the impact of intermittent hyperglycemia on A-FABP expression and the involved inflammatory pathways in macrophages remain unknown.

Considering the notion that fluctuating hyperglycemia may exert more detrimental effects in patients with diabetes or other diseases, the aim of the present study is to compare the effects of persistent and of intermittent hyperglycemia on TLR4 and A-FABP expression and on the inflammatory response in THP-1 macrophages. The present study shows that intermittent hyperglycemia results in higher A-FABP expression and in an increased release of proinflammatory cytokines through TLR4-JNK pathways. These findings strongly indicate that the prevention of fluctuating hyperglycemia is essential to reduce macrophage activation and JNK inhibition may be a potential therapeutic method to inhibit macrophage activation and the resulting inflammatory response.

## 2. Results

### 2.1. Intermittent High Glucose Greatly Induces Upregulation of TLR4 and p-JNK in THP-1 Cells

**Figure 1:**
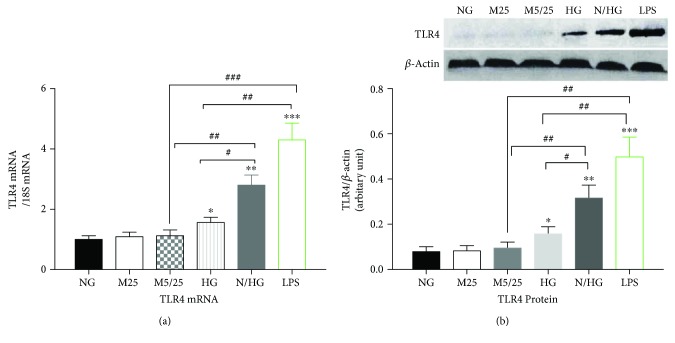
Constant high glucose, intermittent high glucose, and LPS individually induced a robust increase in the expression of TLR4 in THP-1 cells. (a) The mRNA levels of TLR4 were examined 24 h after the treatment of THP-1 cells. (b) The protein levels of TLR4 were examined 24 h after the treatment of THP-1 cells. NG, normal glucose (5 mM); M25, mannitol (25 mM); M5/25, intermittent mannitol (25–5 mM); HG, constant high glucose (25 mM); N/HG, intermittent glucose (25–5 mM). ^∗^*p* < 0.05, ^∗∗^*p* < 0.01, ^∗∗∗^*p* < 0.001 versus ctrl, ^#^*p* < 0.05, ^##^*p* < 0.01, ^###^*p* < 0.001 (*n* = 4 for each group).

As shown in Figure 1, very low expression levels of TLR4 mRNA (Figure 1(a)) and protein (Figure 1(b)) were detected in the constant or intermittent mannitol-treated or normal glucose-treated THP-1 cells. The TLR4 mRNA and protein levels were comparable in these three groups. In contrast, in the persistent presence of 25 mM of glucose, the expression of the TLR4 gene and protein increased as compared with the constant or intermittent mannitol-treated, or normal-glucose group (*p* < 0.05, high-Glu versus 25 mM Man group, versus 5/25 mM Man group, or versus normal-Glu group). However, intermittent high glucose (5 mM glucose for 6 hours and 25 mM glucose for 6 hours per cycle) resulted in more potent upregulation of TLR4 mRNA and protein levels (*p* < 0.01, normal-/high-Glu versus 25 mM Man group, versus 5/25 mM Man group, or versus normal-Glu group). The increased TLR4 expression by fluctuating high glucose was lower than the effect of the TLR ligand LPS, which resulted in a 5–7-fold upregulation of TLR4 mRNA and protein levels in the concentration of 100 ng/ml (*p* < 0.05, normal-/high-Glu group versus LPS group).

**Figure 2:**
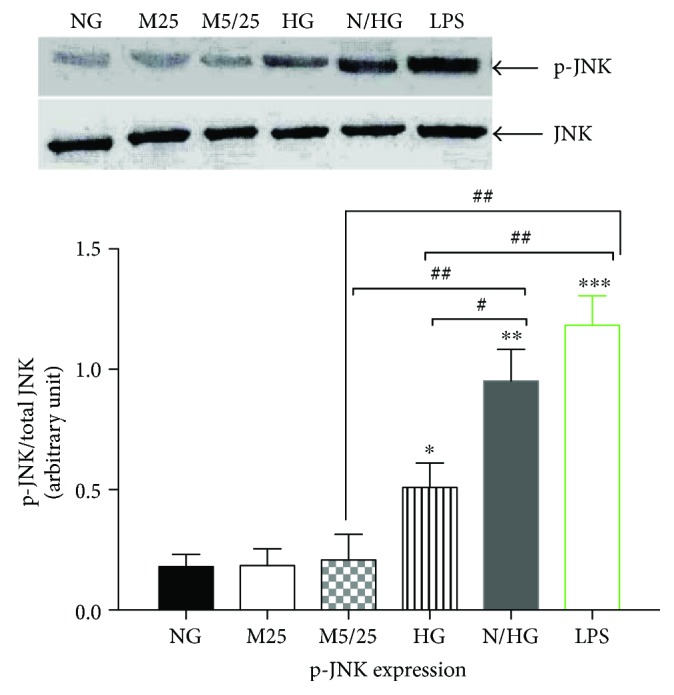
Constant high glucose, intermittent high glucose, and LPS individually increased phosphorylation of JNK in THP-1 cells. NG, normal glucose (5 mM); M25, mannitol (25 mM); M5/25, intermittent mannitol (5–25 mM); HG, constant high glucose (25 mM); N/HG, intermittent glucose (5–25 mM). ^∗^*p* < 0.05, ^∗∗^*p* < 0.01, ^∗∗∗^*p* < 0.001 versus ctrl, ^#^*p* < 0.05, ^##^*p* < 0.01 (*n* = 4 for each group).

As JNK is a downstream signal of TLR4, in the present study we next investigated the effect of high glucose on JNK phosphorylation. As shown in Figure 2, LPS resulted in a more than 6-fold increase in JNK phosphorylation as compared with the constant or intermittent mannitol-treated, or normal-glucose group (*p* < 0.001, LPS group versus 25 mM Man group, versus 5/25 mM Man group, or versus normal-Glu group). Similarly, persistent high glucose led to a milder increase in JNK phosphorylation (*p* < 0.05 high-Glu group versus 25 mM Man group, versus 5/25 mM Man group, or versus normal-Glu group). However, intermittent high glucose activated p-JNK expression to a higher extent as compared with persistent high-glucose treatment (*p* < 0.05, normal-/high-Glu group versus high-Glu group).

### 2.2. Intermittent High Glucose Greatly Upregulates A-FABP Expression and Induces Proinflammatory Cytokine Release

**Figure 3:**
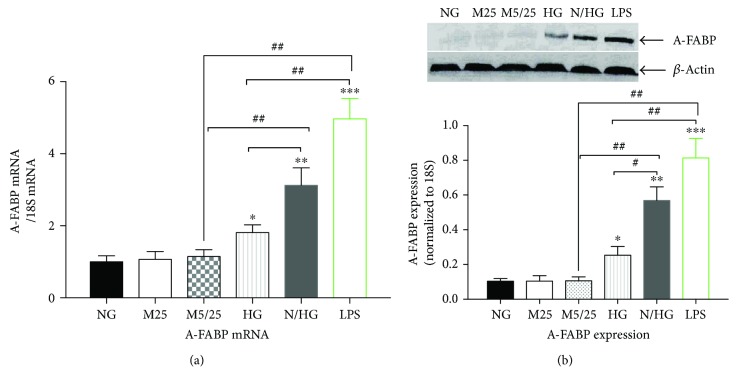
Constant high glucose, intermittent high glucose, and LPS individually induced a robust increase in the expression of A-FABP in THP-1 cells. (a) The mRNA levels of A-FABP were examined 24 h after the treatment of THP-1 cells. (b) The protein levels of A-FABP were examined 24 h after the treatment of THP-1 cells. NG, normal glucose (5 mM); M25, mannitol (25 mM); M5/25, intermittent mannitol (5–25 mM); HG, constant high glucose (25 mM); N/HG, intermittent glucose (5–25 mM). ^∗^*p* < 0.05, ^∗∗^*p* < 0.01, ^∗∗∗^*p* < 0.001 versus ctrl, ^#^*p* < 0.05, ^##^*p* < 0.01 (*n* = 4 for each group).

Our previous studies have shown that LPS induces A-FABP activation and the following release of cytokines through the TLR4-JNK pathway [21]. Consistent with the previous study, LPS (100 ng/ml) dramatically increased the expression of A-FABP mRNA and protein (*p* < 0.001, LPS group versus 25 mM Man group, versus 5/25 mM Man group, or versus normal-Glu group) (Figure 3). Persistent high glucose also induced the upregulation of A-FABP to a much lower extent than LPS treatment (*p* < 0.01, high-Glu group versus LPS group). In contrast, intermittent high glucose caused a more robust upregulation of A-FABP as compared with persistent high glucose (*p* < 0.05 normal-/high-Glu group versus high-Glu group), but less than that of LPS treatment (*p* < 0.05, normal-/high-Glu group versus LPS group).

**Figure 4:**
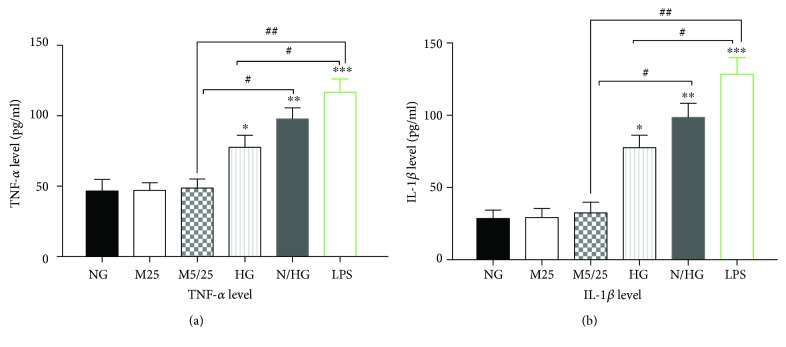
Constant high glucose, intermittent high glucose, and LPS individually increased the production of inflammatory cytokines TNF-*α* and IL-1*β* in THP-1 cells. (a) The TNF-*α* levels were determined 24 h after the treatment of THP-1 cells. (b) The IL-1*β* levels were determined 24 h after the treatment of THP-1 cells. NG, normal glucose (5 mM); M25, mannitol (25 mM); M5/25, intermittent mannitol (5–25 mM); HG, constant high glucose (25 mM); N/HG, intermittent glucose (5–25 mM). ^∗^*p* < 0.05, ^∗∗^*p* < 0.01, ^∗∗∗^*p* < 0.001 versus ctrl, ^#^*p* < 0.05, ^##^*p* < 0.01 (*n* = 4 for each group).

Like the activation of A-FABP, LPS treatment remarkably increased the supernatant TNF-*α* and IL-1*β* levels (Figures 4(a) and 4(b)) (*p* < 0.001, LPS group versus 25 mM Man group, versus 5/25 mM Man group, or versus normal-Glu group). Be it to a lower extent, persistent or intermittent high glucose also increased the supernatant levels of TNF-*α* (Figure 4(a)) and IL-1*β* (Figure 4(b)). However, intermittent high glucose increased the levels of these two cytokines more than persistent high-glucose treatment did (*p* < 0.05, normal-/high-Glu group versus high-Glu group).

### 2.3. JNK Inhibitor SP600125 Did Not Attenuate the Activation of TLR4 by Persistent and Intermittent High Glucose

**Figure 5:**
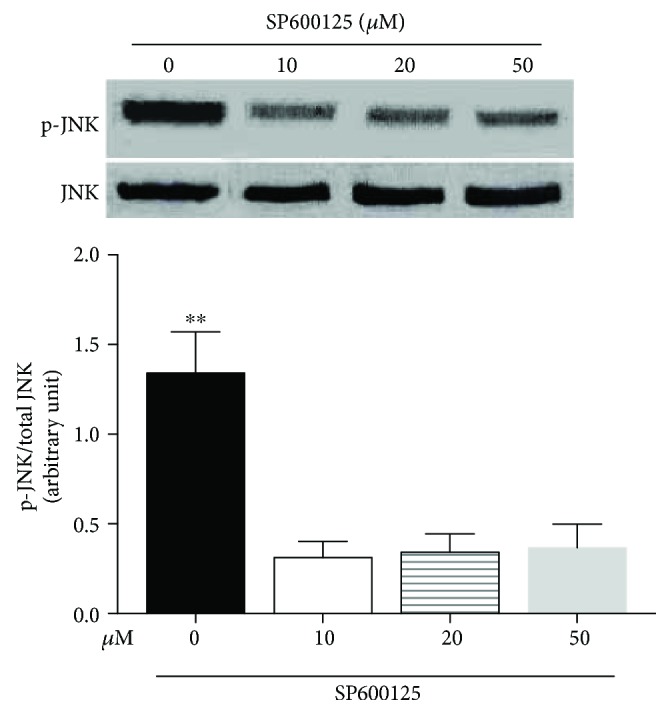
SP600125 treatment at different concentrations downregulated the increased JNK phosphorylation induced by LPS treatment. The levels of JNK phosphorylation in THP-1 cells pretreated with 10 *μ*M, 20 *μ*M, or 50 *μ*M SP600125 for 2 h followed by stimulation with LPS. ^∗∗^*p* < 0.01, 0 *μ*M group versus 10 *μ*M group, versus 20 *μ*M group, or versus 50 *μ*M group (*n* = 4 for each group).

To further explore the role of TLR4-JNK pathways on the activation of A-FABP and the following inflammatory response, the JNK inhibitor SP600125 was used to inhibit JNK phosphorylation. As shown in Figure 5, all three concentrations of SP600125 tested in the present study (10 *μ*M, 20 *μ*M, and 50 *μ*M) almost completely prevented the increased JNK phosphorylation induced by LPS treatment (*p* < 0.01, 0 *μ*M group versus 10 *μ*M group, versus 20 *μ*M group, or versus 50 *μ*M group), suggesting that SP600125 is a potent inhibitor of JNK activity.

**Figure 6:**
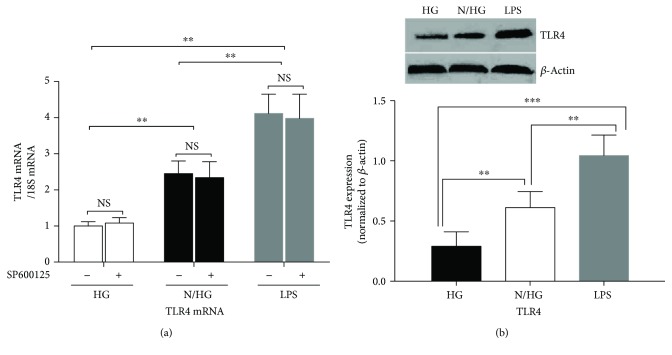
JNK inhibitor SP600125 did not inhibit the activation of TLR4 by persistent or intermittent high glucose. THP-1 cells were pretreated with SP600125 (20 *μ*M) for 2 h followed by stimulation with intermittent high glucose, persistent high glucose, or LPS. Then the expression of TLR4 was examined. ^∗∗^*p* < 0.01, ^∗∗∗^*p* < 0.001 (*n* = 4 for each group).

In sharp contrast, SP600125 treatment (20 *μ*M) did not inhibit the upregulation of TLR4 mRNA induced by intermittent high glucose or LPS treatment (Figure 6(a)) (*p* < 0.05, normal-/high-Glu group versus high-Glu group; *p* < 0.01, LPS group versus high-Glu group). Similarly, the upregulation of TLR4 induced by intermittent high glucose or LPS treatment was not changed after SP600125 treatment (Figure 6(b)).

### 2.4. SP600125 Inhibited the Activation of A-FABP and Reduced the Release of TNF-*α* and IL-1*β* Induced by Persistent and Intermittent High Glucose

**Figure 7:**
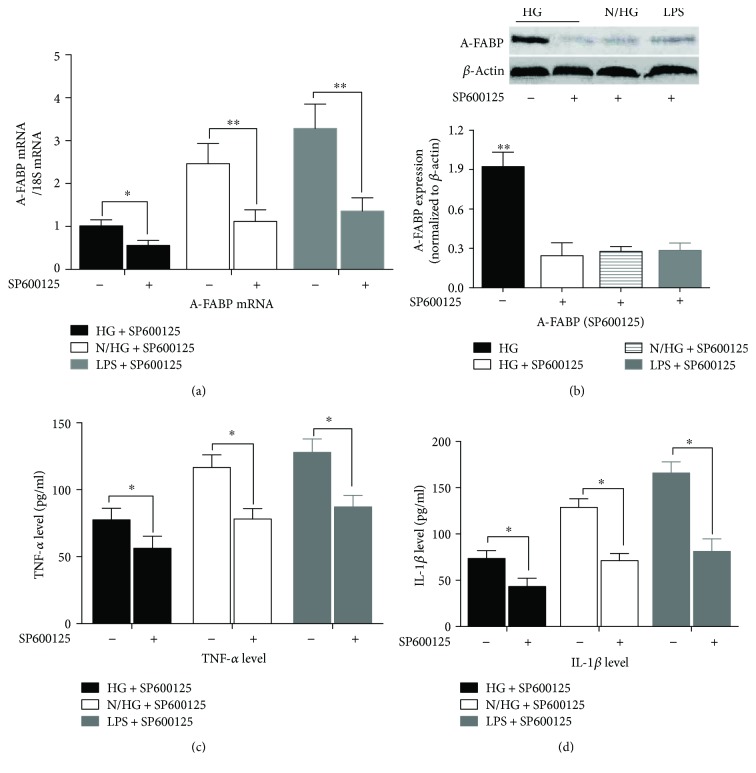
SP600125 (20 *μ*M) treatment greatly inhibited the production of A-FABP and inflammatory cytokines induced by intermittent high glucose or LPS treatment. (a) The levels of A-FABP mRNA and (b) A-FABP protein were analyzed in THP-1 cells pretreated with 20 *μ*M SP600125 for 2 h followed by stimulation with glucose or LPS. (c) The release of TNF-*α* in THP-1 cells pretreated with SP600125 followed by stimulation with glucose or LPS. (d) Effects of SP600125 on the production of IL-1*β* in THP-1 cells after glucose or LPS treatment for 24 h. ^∗^*p* < 0.05, ^∗∗^*p* < 0.01 (*n* = 4 for each group).

In the present study, we next investigated the effect of SP600125 on the high-glucose-evoked upregulation of A-FABP and the increased TNF-*α* and IL-1*β* supernatant levels. As shown in Figure 7(a), in the presence of 20 *μ*M SP600125, A-FABP mRNA levels in high-Glu, normal-/high-Glu, and LPS groups were much lower than their match groups without SP600125 treatment, respectively (*p* < 0.05, high Glu + SP600125 versus high Glu; *p* < 0.01, normal/high Glu + SP600126 versus normal/high Glu and LPS + SP600125 versus LPS).  Figure 7(b) shows the dramatic inhibition of A-FABP protein expression after SP600125 treatment (*p* < 0.01, high Glu versus high Glu + SP600125, or versus normal/high Glu + SP600125, or versus LPS). These results suggest that SP600125 remarkably inhibits A-FABP activation upon the treatment of persistent or intermittent high glucose.

Figures 7(c) and 7(d) show that SP600125 greatly inhibits the release of TNF-*α* (Figure 7(c)) and IL-1*β* (Figure 7(d)) induced by the persistent or intermittent high glucose (*p* < 0.05, high Glu + SP600125 versus high Glu and normal/high Glu + SP600125 versus normal/high Glu). Notably, the inhibitory effect on TNF-*α* and IL-1*β* release of SP600125 was milder than its inhibitory effect on A-FABP expression (Figures 7(a) and 7(b)).

## 3. Discussion

Glucose level variability is emerging as an important index of diabetes control, even though its underlying mechanisms on diabetic complications and the immune system are only beginning to be elucidated. The current study shows that both consistent and intermittent hyperglycemia increase A-FABP mRNA and protein expression, in parallel with the production of proinflammatory cytokines TNF-*α* and IL-1*β*, the upregulation of which is mediated by upregulated phosphorylation of JNK in response to high or fluctuating glucose. However, intermittent hyperglycemia appeared to worsen the aberrant production of A-FABP and the proinflammatory effects of high glucose on THP-1 cells.

A growing body of literature has revealed the close involvement of chronic low-grade inflammation and of the innate immune system in the development of diabetes and its vascular complications. In epidemiologic studies, a variety of circulating inflammatory markers, such as IL-1*β*, have been identified as strong predictors of type 2 diabetes [24–26]. Moreover, monocytes isolated from patients with type 1 diabetes also present a proinflammatory phenotype and secrete higher levels of inflammatory cytokines, such as IL-6 and IL-1*β*, compared with nondiabetic individuals [27]. Hyperglycemia can cause an M_1_/M_2_ imbalance, switching macrophage polarization towards a proinflammatory M_1_ phenotype [12, 28–31]. In the present study, we have also detected that high glucose increases the level of TNF-*α* and IL-1*β*, markers of M_1_ macrophages, confirming that high glucose induces the inflammatory response in macrophages. More importantly, intermittent high glucose caused a more robust upregulation of TNF-*α* and IL-1*β*. These findings strongly indicate that, as compared with persistent high glucose, violent fluctuation of glucose exerts more detrimental inflammatory responses in patients.

Most studies on diabetes-related diseases were performed with treatment of consistent high glucose levels. These studies did not mimic an actual clinical context, where most diabetic patients experience violent fluctuations of blood glucose levels every day. Indeed, glucose oscillation is equally important as HbA_1_c in the control of diabetes and it has been reported to be a good predictor for the development of diabetic complications in a growing body of literature. However, compared with numerous studies on the impact of consistent hyperglycemia on macrophages, there is little knowledge available on the questions whether intermittent hyperglycemia causes aberrant activation of macrophages and whether there is a difference in the extent of inflammation between cases of consistent and intermittent hyperglycemia. The more severe inflammatory responses likely to be caused by intermittent hyperglycemia have been reported in monocytes and umbilical endothelial cells [32, 33]. Notably, in the previous study of monocytes, the author also used THP-1 cells, but merely stimulated differentiation into the monocyte form of THP-1 cells under high glucose conditions, without the addition of PMA for differentiation into macrophages [33]. Indeed, after reaching the tissue, monocytes differentiate into macrophages, which play a number of critical roles in diabetes, atherosclerosis, and other health issues. Because macrophages and monocytes exhibit distinct phenotypes, we further looked into the responses of macrophages to intermittent and consistent high glucose. Compared with a previous study in monocytes [33], both consistent and intermittent hyperglycemia increased a more dramatic production of the inflammatory cytokines TNF-*α* and IL-1*β* in macrophages, and this proinflammatory effect is more pronounced in response to intermittent hyperglycemia. This finding further supports the deleterious effects of glucose fluctuation on macrophage inflammation in micro- and macrodiabetic complications.

The present study has mimicked glucose toxicity under consistent and intermittent high glucose conditions to clarify the relationship between A-FABP and glucose toxicity in the control of macrophage inflammation. Hyperglycemia is shown to induce inflammation through different signaling cascades, including (i) Rho-associated coiled-coil forming kinase (ROCK)-dependent JNK and extracellular regulated protein kinase (ERK) phosphorylation in RAW264.7 macrophages [28], (ii) signal transducer and activator of transcription 3 (STAT3) in retinal endothelial cells [34], and (iii) TLR2 and TLR4 in human microvascular retinal endothelial cells [35]. LPS activates the JNK activator protein-1 (AP-1) signaling pathway and leads to the upregulation of A-FABP and the release of proinflammatory cytokines in macrophages. Consistently, we have shown that p-JNK is activated by consistent or intermittent high glucose; pharmaceutical inhibition of p-JNK completely abolished glucose-induced A-FABP expression and the production of the inflammatory cytokines TNF-*α* and IL-1*β*. This indicates that p-JNK is involved in hyperglycemia-induced macrophage inflammation.

Our data also show that A-FABP levels increase during the inflammatory response induced by consistent or intermittent hyperglycemia in macrophages. A-FABP belongs to the fatty acid-binding protein family and has been identified as a critical mediator linking diabetes and cardiovascular diseases, partly through its proinflammatory actions in macrophages [16]. Mice deficient in FABP4 and FABP5 reveal a striking phenotype with strong protection against diet-induced obesity, insulin resistance, type 2 diabetes and fatty liver disease [36]. However, the influence of high glucose, consistent or intermittent, on the expression of A-FABP in macrophages remains unclear. In our experiments, the exposure of THP-1 cells to high glucose concentrations induced an increase in mRNA and protein expression of A-FABP, which was higher in intermittent high glucose than in consistent high glucose. In line with our results, Yao et al. treated human mesangial cells with stable high glucose (30 mM) for 24 hours, which dramatically increased A-FABP mRNA and protein expression. Consistent with our findings, a close connection between A-FABP and p-JNK has been reported in the LPS-evoked inflammatory response. After the exposure of RAW264.7 cells to LPS, activated JNK increases A-FABP mRNA and protein expression through the binding of a phosphorylated site of c-Jun to a highly conserved AP-1 *cis*-element within the A-FABP gene promoter [21]. It should be noted that many diabetes patients are subjected to infection or sepsis, in which the TLR4 pathway may be activated to a certain extent. Our study suggests that, under this condition, the avoidance of fluctuating high glucose levels is more important to control the strong inflammatory response than lowering blood glucose levels.

## 4. Conclusion

In summary, this study shows that the exposure of THP-1 cells to intermittent high glucose results in a more robust overproduction of A-FABP and proinflammatory cytokines than stable high glucose, which is mediated by the TLR4/p-JNK signaling cascade. These findings suggest a more ominous impact of glucose fluctuation on macrophage functioning in diabetes-related vascular diseases and strongly imply that prevention of violent glucose fluctuations is critical for the delay of diabetes-related diseases.

## 5. Materials and Methods

### 5.1. Cell Culture and Treatment

THP-1 human monocytic cells (Shanghai Institute of Cell Biochemistry and Cell Biology, Chinese Academy of Sciences, Shanghai, China) were cultured in RPMI-1640 medium (Invitrogen, Carlsbad, USA) supplemented with 10% FBS and 1% P/S, at 37°C under 5% CO_2_. The cells were differentiated to macrophages by treatment with 100 nM phorbol-12-myristate-13-acetate (PMA) (Sigma-Aldrich, St. Louis, MO, USA) in RPMI-1640 supplemented with 10% FBS and 1% P/S for 72 h. After 72 h of differentiation, cells were divided into four groups and incubated for 24 h as follows: (1) constant 5 mM glucose, (2) constant 25 mM glucose, (3) alternating normal (5 mM) and high (25 mM) glucose medium every 6 h, and (4) 100 ng/ml LPS. Osmotic control was assured by treating THP-1 cells with equimolar concentrations of mannitol (Sigma-Aldrich, St. Louis, MO), both continuously and in an alternating manner.

### 5.2. Real-Time Polymerase Chain Reaction

Real-time polymerase chain reaction (PCR) was performed to detect the gene expression of A-FABP, TLR4, and *β*-actin. The PCR sequence pairs were: AACCTTAGATGGGGGTGTCC and ATGCGAACTTCAGTCCAGGT (A-FABP); GCCGACAGGATGCAGAAGGAG and AAGCATTTGCGGTGGACGATG (*β*-actin); AAGCCGAAAGGTGATTGTTG and CTGAGCAGGGTCTTCTCCAC (TLR4). PCR reaction was carried out using 15 ng of cDNA, 200 nM of each primer and SYBR green PCR master mix (Roche, Germany). Cycling conditions included 10 min at 95°C followed by 40 cycles of 15 s at 95°C and 60 s at 60°C.

### 5.3. Western Blotting Analysis

After treatment, protein extracts for total cellular fractions were isolated in a cell lysis buffer (Cell Signaling Technology, Beverly, MA, USA) supplemented with 0.1 mg PMSF and a 1/100 dilution of protease and phosphatase inhibitor cocktails (Sigma-Aldrich, St. Louis, MO). Scraped samples were then centrifuged at 10000 rpm for 15 min at 4°C. The supernatants were used for Western blotting. Protein concentrations were measured using the Bio-Rad protein dye microassay (Bio-Rad, Hercules, USA). Proteins were separated on a 12% SDS-PAGE gel and then transferred onto PVDF membranes. The membrane was blocked with a solution of TBS and 5% fat-free milk for 1 h, then incubated overnight with rabbit anti-A-FABP, rabbit anti-phospho-JNK, rabbit anti-JNK, rabbit anti-TLR4 (all from Cell Signaling Technology, Beverly, MA, USA), or monoclonal mouse anti-*β*-actin (Santa Cruz Biotechnology, Heidelberg, Germany). The blot was then incubated with horseradish peroxidase-conjugated goat anti-rabbit IgG or goat anti-mouse IgG in TBS for 2 h at room temperature. The membrane was then exposed to film before development.

### 5.4. TNF-*α* and IL-1*β* Quantification

Conditioned media from cultured cells were collected at the indicated time points. The quantitative detection of soluble TNF-*α* and IL-1*β* was determined using commercially available ELISA kits (BioSource International, USA), in accordance with the manufacturer's instructions.

### 5.5. Statistical Analysis

Data are expressed as mean ± standard error of the mean (SEM). Statistical analyses were performed using the paired Student's *t* test or one-way analysis of variance (ANOVA) followed by Bonferroni analysis where appropriate. Statistical significance was arbitrarily declared at *p* values below 0.05. All analyses were performed using SPSS version 20 (SPSS Inc., Chicago, IL).
